# Supplementary material for: The interactome of intact mitochondria by cross-linking mass spectrometry provides evidence for coexisting respiratory supercomplexes
Source: Mol Cell Proteomics. 2017 Dec 8;17(2):216–32. doi: 10.1074/mcp.RA117.000470 (PMC5795388; doi:10.1074/mcp.RA117.000470)
Supplement: Supplemental Data [file supp_RA117.000470_133922_0_supp_23978_fzffwf.zip › spectra_annotation/mito_spectra_annotation/321-1-14-1-11-1.pdf]

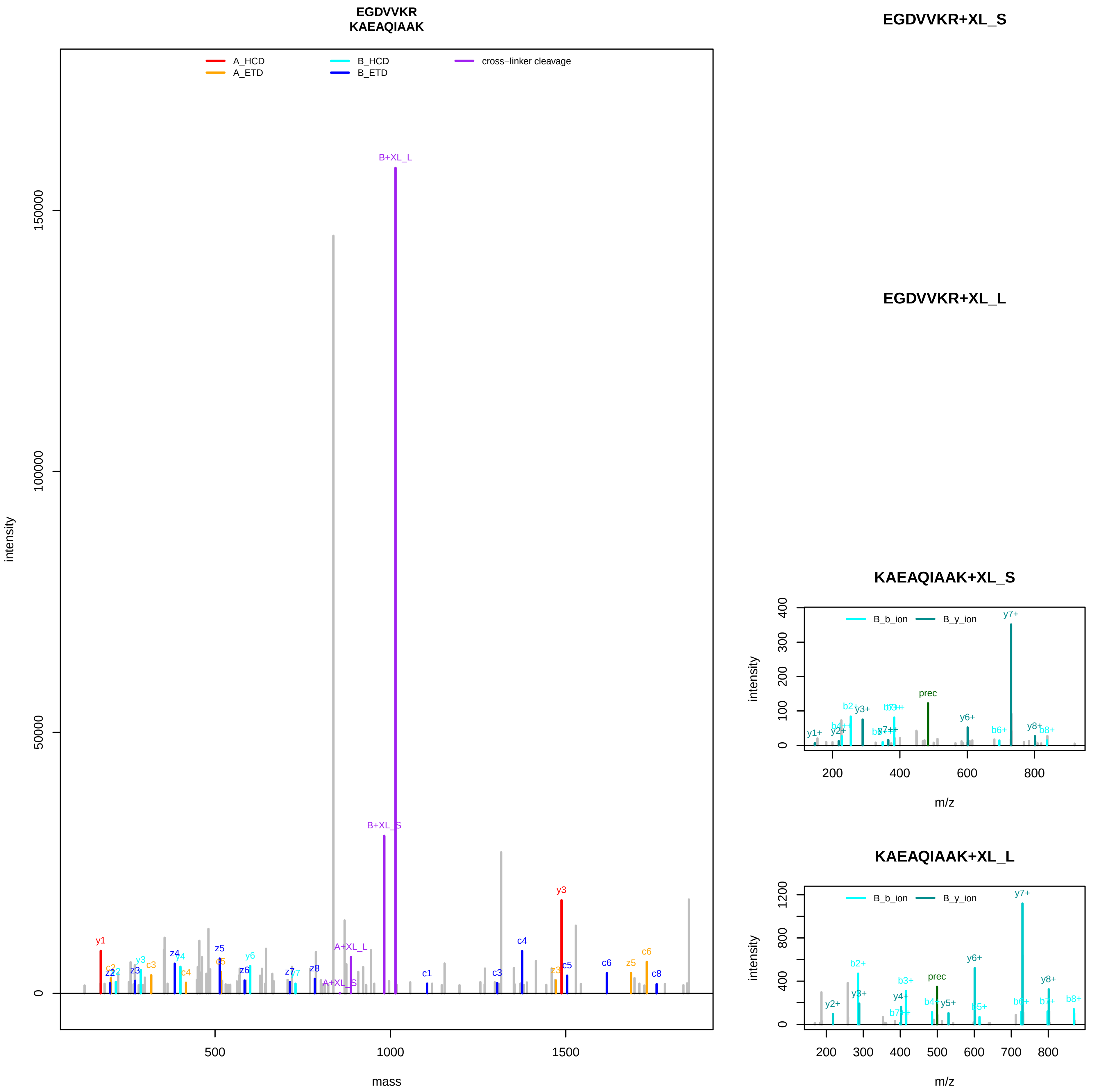
EGDVVKR
KAEAQIAAK
A_HCD
A_ETD
B_HCD
B_ETD
cross−linker cleavage
B+XL_L
B+XL_S
A+XL_L
A+XL_S
y1
y3
c6
z5
c8
c1
c3
c4
c5
c6
z4
z5
z6
z7
z8
y6
intensity
mass
0
50000
100000
150000
500
1000
1500
EGDVVKR+XL_S
EGDVVKR+XL_L
KAEAQIAAK+XL_S
B_b_ion
B_y_ion
y7+
prec
y6+
b6+
y8+
b8+
b2+
y3+
y1+
y2+
intensity
m/z
0
100
200
300
400
200
400
600
800
KAEAQIAAK+XL_L
B_b_ion
B_y_ion
y7+
y6+
y8+
b2+
b3+
y3+
y4+
prec
b4+
y5+
b5+
b6+
b7+
b8+
y2+
intensity
m/z
0
400
800
1200
200
300
400
500
600
700
800
